# A pre-specified model based on four kallikrein markers in blood improves predictions of adverse pathology and biochemical recurrence after radical prostatectomy

**DOI:** 10.1038/s41416-020-0914-7

**Published:** 2020-05-29

**Authors:** Alexander Haese, Amy L. Tin, Sigrid V. Carlsson, Daniel D. Sjoberg, Dirk Pehrke, Thomas Steuber, Hartwig Huland, Markus Graefen, Peter T. Scardino, Thorsten Schlomm, Andrew J. Vickers, Hans Lilja, Guido Sauter

**Affiliations:** 1 Martini-Klinik Prostate Cancer Center, University Medical Center Hamburg-Eppendorf, Hamburg, Germany; 2 Department of Epidemiology and Biostatistics, Memorial Sloan Kettering Cancer Center, New York, NY USA; 3 Department of Surgery (Urology Service), Memorial Sloan Kettering Cancer Center, New York, NY USA; 4 Department of Urology, Institute of Clinical Sciences, Sahlgrenska Academy at University of Gothenburg, Gothenburg, Sweden; 5 Department of Urology, Charité - Universitätsmedizin Berlin, Berlin, Germany; 6 Departments of Laboratory Medicine and Medicine, Memorial Sloan Kettering Cancer Center, New York, NY USA; 7 Nuffield Department of Surgical Sciences, University of Oxford, Oxford, UK; 8 Department of Translational Medicine, Lund University, Malmö, Sweden; 9 Institute of Pathology, University Medical Center Hamburg-Eppendorf, Hamburg, Germany

**Keywords:** Cancer screening, Prostate cancer, Predictive markers

## Abstract

**Background:**

A pre-specified model based on four kallikrein markers in blood, commercially available as 4Kscore, predicts Gleason Grade (GG) 3 + 4 or higher prostate cancer on biopsy. However, sampling error and variation in pathology reporting may miss aggressive disease.

**Methods:**

The 4Kscore was measured in cryopreserved blood from 2330 men obtained before prostatectomy at a single institution between 2002 and 2010. Adverse surgical pathology and biochemical recurrence (BCR) were pre-specified to be assessed in all men, biopsy GG 3 + 3, and 3 + 4.

**Results:**

Adjusted for established clinical predictors, the 4Kscore was significantly associated with adverse pathology (OR 1.49; 95% CI 1.32, 1.67; *p* < 0.0001). Adding 4Kscore increased discrimination from (AUC) 0.672 to 0.718 and 0.644 to 0.659 within biopsy GG 3 + 3 and 3 + 4, respectively. Higher 4Kscore was associated with higher risk of BCR (HR 1.16, 95% CI 1.06, 1.26; *p* = 0.001). Adding 4Kscore improved the prediction of BCR (C-index 0.630–0.660) within GG 3 + 3, but not GG 3 + 4.

**Conclusions:**

The 4Kscore can help guide the clinical decision whether additional risk assessment—such as confirmatory biopsy—is needed to decide between active surveillance versus curative therapy. Evidence that the panel could influence management in biopsy GG 3 + 4 is less strong and requires further investigation.

## Background

Measurement of PSA in blood is the most common method to screen men for prostate cancer, and has been shown to reduce prostate cancer mortality. However, with its low specificity, most men with moderately elevated PSA do not have aggressive prostate cancer.^1^ Prostate biopsy is an invasive and uncomfortable diagnostic procedure associated with non-trivial risks of complications, including rectal haemorrhage, urinary tract infection, sepsis and hospitalisation.^2,3^ Moreover, the use of liberal criteria for biopsy are associated with the risk of identifying low-grade prostate cancer, which not only leads to the expense, inconvenience and anxiety of active surveillance, but often leads to overtreatment. Developing methods to improve the specificity and reduce the downstream harms of the PSA test is a major public health priority.

If low-to-intermediate-risk cancer (Gleason 3 + 3 and 3 + 4) is found on biopsy, the urologist faces challenging clinical decisions: (i) for those with Gleason 3 + 3, whether or not to perform a confirmatory biopsy or other risk assessment before recommending active surveillance,^4,5^ and (ii) for those with Gleason 3 + 4, whether or not to recommend curative treatment—surgery or radiation.

Prostate biopsy involves sampling the prostate and may underestimate disease severity. Approximately 30–40% of men with Gleason 3 + 3 on initial biopsy will have higher-grade cancer in the prostatectomy specimen.^6–8^ More accurate assessment of the nature of the cancer would increase both physician and patient confidence in the safety of active surveillance, or indications for immediate treatment.^9,10^

A statistical model based on a panel of 4K markers in blood-total, free, intact PSA and hK2, commercialised by OPKO Health Inc. (Miami, FL, USA) as the 4Kscore test can accurately predict Gleason 3 + 4 or higher prostate cancer on biopsy.^11–14^ The 4Kscore has also been shown to predict prostate cancer death in men followed for many years without screening.^15,16^ This suggests that the 4Kscore might aid risk stratification in patients with low- and intermediate-risk cancer on biopsy. Our objective was to assess the ability of the 4Kscore to predict adverse pathology at prostatectomy—the gold standard for accurate histological diagnosis—and BCR, with a focus on men diagnosed with Gleason 3 + 3 or 3 + 4 prostate cancer at biopsy.

## Methods

### Study design

This retrospective study included 2330 men with localised prostate cancer undergoing radical prostatectomy at Martini-Klinik in Hamburg, Germany, a tertiary referral centre, between 2002 and 2010. All biopsies were 10–12-core transrectal ultrasound-guided biopsy using a standard template, with biopsy and pathological evaluation conducted at the Martini-Klinik. Kallikrein markers were measured in preoperative blood cryopreserved at −80 °C. The rate of active surveillance at the time was very low, with almost all patients treated shortly after diagnosis. We excluded patients with missing pathology data at prostatectomy (*n* = 22), missing kallikrein measurements (*n* = 5) and suspected non-specific analytical interference in kallikrein measurements (*n* = 3).

### Test methods

Sample aliquots were shipped to Dr. Lilja’s laboratory at Lund University in Malmö, Sweden for measurements of kallikrein levels conducted in 2016–2017 blind to outcome. Total and free PSA levels were measured using the AutoDelfia 1235 automatic immunoassay system using the dual-label DELFIA Prostatus total/free PSA-Assay (Perkin-Elmer, Turku, Finland) calibrated against the World Health Organization (WHO) 96/670 (PSA-WHO) and WHO 68/668 (free PSA-WHO) standards. Intact PSA and hK2 were measured with F(ab’)2 fragments of the monoclonal capture antibodies to reduce the frequency of non-specific assay interference, as described in detail previously.^17,18^ To reduce interobserver variability of pathologic specimen, all Gleason Grade 3 + 3 and 3 + 4 biopsies and prostatectomy specimens were read at the Institute of Pathology of the University Clinic Hamburg Eppendorf. Markers were assayed blind to clinical outcome and vice versa.

### Statistical methods

Adverse pathology at prostatectomy was defined by Brand et al.:^19^ primary Gleason pattern 4, any pattern 5 or non-organ-confined disease: seminal vesicle invasion, ECE or lymph node invasion. BCR was defined as a PSA level ≥0.20 ng/mL.

Logistic and Cox regression were used to study the association between 4Kscore and adverse pathology and BCR, respectively. Discrimination was assessed by the AUC for adverse pathology and C-index for BCR, comparing the improvement in discrimination by adding 4Kscore to a preoperative clinical base model.

We first defined a clinical logistic model—age at blood draw, total PSA, biopsy Gleason Grade (3 + 3 vs 3 + 4 vs 4 + 3 vs >4 + 3), and clinical tumour stage (<T2b vs ≥T2b)—to predict adverse pathology. Next, we calculated the 4Kscore—using the pre-specified formula developed in the ProtecT cohort^20^—for each patient. The 4Kscore-only model was defined using a univariate logistic model to predict adverse pathology. Lastly, we defined a full model by including both 4Kscore and the variables in the clinical model, to predict adverse pathology. For all models, the logit transformation of the 4Kscore was used. Since patients with blood sample available prior to surgery were not representative of the distribution of all radical prostatectomy patients at Martini-Klinik, with a larger proportion having lower Gleason Grade, all logistic models included sampling weights equal to the inverse of the probability of patients with blood sample available based on Gleason Grade.

To confirm whether the 4Kscore offers additional predictive ability after adjusting for the clinical model, we reported the estimates for the 4Kscore from the full model. The predictive accuracy of the clinical model, 4Kscore-only model and the full model was ascertained by calculating bootstrapped (using 200 bootstrap samples) optimism-corrected AUC, pre-specified to be assessed in the two groups of men where a clinical decision needs to be made: biopsy Gleason Grade 3 + 3 and 3 + 4, separately. Clinical utility was assessed using decision-curve analysis.^21^

Sensitivity analyses included (i) using a more restrictive definition of adverse pathology with ECE excluded from the definition, (ii) defining the clinical model to include the number of positive cores and millimetres of cancerous tissue on biopsy, (iii) excluding patients with low 4Kscores who may have never had been biopsied and diagnosed, had they received a 4Kscore, (iv) incorporating transrectal ultrasound volume into the clinical models and (v) excluding radical prostatectomy cases from 2002 to 2004, which were graded prior to the 2005 ISUP Modified Gleason System, and therefore patients considered to have pattern 3 disease may be regraded to pattern 4 on the modern grading system. All possible combinations of adverse pathology, clinical models and subgroups as defined above, were assessed.

To assess the association between 4Kscore and BCR, we excluded 195 patients with missing data on recurrence, and 71 men who underwent adjuvant treatment, defined as any additional treatment within 6 months of surgery. Twenty-six men who underwent salvage treatment prior to the recorded date of BCR were considered to have had BCR at the time of treatment. Among the remaining 2064 patients, we used a univariable Cox regression model to assess the association, then created two multivariable Cox models to ascertain whether 4Kscore offered additional predictive ability after adjusting for a preoperative prediction model (PSA, clinical stage and biopsy Gleason Grade) and post-operative prediction model (PSA, Gleason Grade on pathology, ECE, seminal vesicle invasion, lymph node invasion and surgical margin status). We then assessed the association between 4Kscore and BCR in the preoperative setting among men with biopsy Gleason Grade 3 + 3 and 3 + 4, separately, and evaluated the discriminative accuracy by calculating the change in the C index when including 4Kscore. An exploratory analysis assessing whether the association between 4Kscore and outcomes differed based on the expression of five molecular markers—ERG, PTEN, EZH2, FOXA1 and HOXB13—in biopsy tissue is described in full in the supplementary material with distribution of the molecular markers shown in Supplementary Table 1 and the results shown in Supplementary Tables 2–5. All statistical analyses were conducted using STATA 15.0 (StataCorp, College Station, TX, USA) and R version 3.5.1 (R foundation for Statistical Computing, Vienna, Austria).

## Results

Patient characteristics are displayed in Table 1. The median age at blood draw was 64 years (IQR 59, 67). Nearly two-thirds of men who underwent prostatectomy had Gleason 3 + 3 cancer at biopsy. A total of 709 men (30%) were found to have adverse pathology. The estimated rate of adjuvant treatment in this cohort was 21%.

**Table 1:** Patient and tumour characteristics.

	*N* = 2330
Age at blood draw	64 (59, 67)
Total PSA	6.0 (4.3, 8.9)
Free PSA	0.94 (0.64, 1.40)
Intact PSA	0.48 (0.33, 0.73)
Days from blood draw to biopsy	0 (−39, 0)
Days from blood draw to radical prostatectomy	1 (1, 3)
hK2—kallikrein-related peptidase 2	0.08 (0.05, 0.11)
Biopsy Gleason Grade	
3 + 3	1484 (64%)
3 + 4	524 (22%)
4 + 3	200 (8.6%)
>4 + 3	122 (5.2%)
Pathologic Gleason Grade	
3 + 3	963 (41%)
3 + 4	1054 (45%)
4 + 3	256 (11%)
>4 + 3	57 (2.4%)
Extracapsular extension	430 (18%)
Seminal vesicle invasion	167 (7.2%)
Lymph node invasion	73 (3.1%)
Favourable pathology	
pT2, N0, Pathologic Gleason Grade 3 + 3	914 (39%)
pT2, N0, Pathologic Gleason Grade 3 + 4	707 (30%)
Adverse pathology	
pT2, N0, Pathologic Gleason Grade ≥4 + 3	110 (4.7%)
pT2, N1	2 (<0.1%)
pT3a, N0	405 (17%)
pT3a, N1	25 (1.1%)
pT3b–pT4, N0	121 (5.2%)
pT3b–pT4, N1	46 (2.0%)
Number of positive cores (*N* = 1798)	2 (1, 4)
Prostate volume on TRUS (*N* = 2261)	44.0 (33.0, 58.3)

All values are median (IQR) or frequency (proportions).

**Fig. 1:**
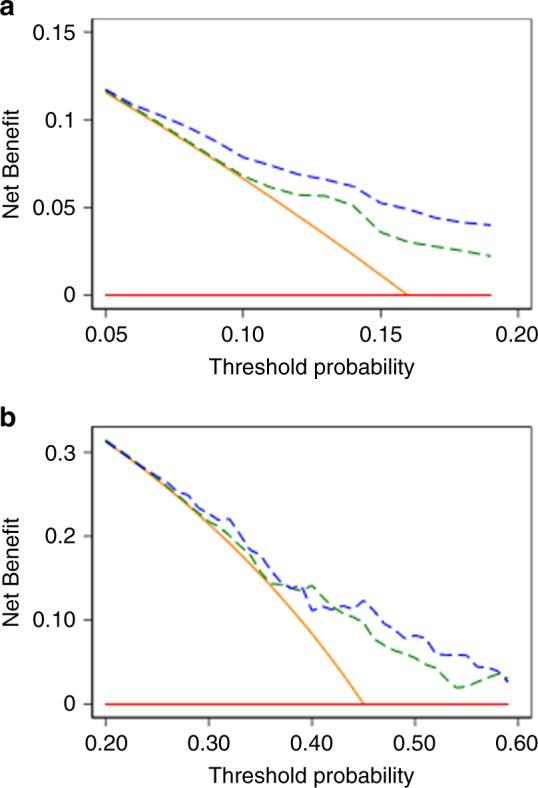
Decision curve analysis based on Gleason Grade. The decision curve analysis compares the net benefit of the clinical + 4Kscore model (blue or dark grey dashed line), clinical-model (green or light grey dashed line), treat-all (orange or light grey solid line), and treat-none (horizontal red or grey solid line) strategies among biopsy (**a**) Gleason Grade 3 + 3 patients and (**b**) Gleason Grade 3 + 4 patients.

**Fig. 2:**
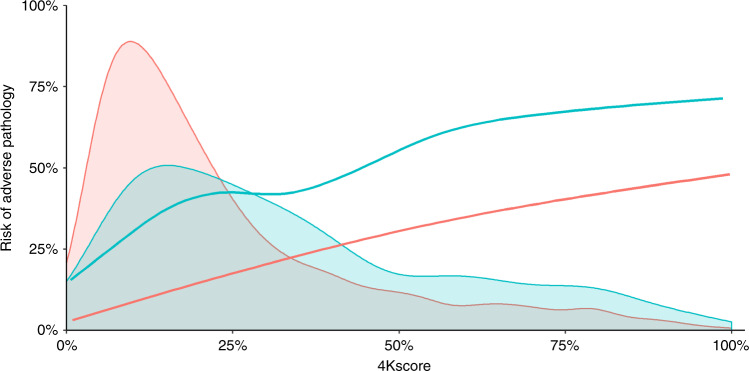
Risk of adverse pathology and distribution of 4Kscore based on Gleason Grade. Risk of adverse pathology against 4Kscore for men with Gleason Grade 3 + 3 (grade group 1, *n* = 1484; pink or light grey) or Gleason Grade 3 + 4 (grade group 2, *n* = 524; blue or dark grey) disease on biopsy, overlaid on the distribution of 4Kscore in the respective Gleason Grade. For instance, a man with biopsy Gleason Grade 3 + 4 cancer and a predicted risk of 10% has about a 30% risk of adverse pathology.

On multivariable regression, 4Kscore was significantly associated with adverse pathology, after adjusting for clinical variables (OR 1.49; 95% CI 1.32, 1.67; *p* < 0.0001; Table 2). The results are shown in Table 2, with all sensitivity analyses shown in Supplementary Table 6a–6d. The optimism-corrected AUC for the clinical model was 0.672 and 0.644 among patients with biopsy Gleason 3 + 3 and 3 + 4, respectively. Adding 4Kscore to the clinical model increased the AUC to 0.718 and 0.659, respectively (Table 2). Sensitivity analyses did not importantly change these findings, with clear evidence of benefit in biopsy Gleason 3 + 3 patients and smaller and less consistent benefit in biopsy Gleason 3 + 4 disease (Table 2 and Supplementary Table 7a–7d). Decision-curve analysis illustrated the improvement in net benefit of the 4Kscore in appropriate ranges for a decision threshold of 5–20% for biopsy Gleason 3 + 3, and to a lesser extent, 20–60% for biopsy Gleason 3 + 4 (Fig. 1a, b). To better illustrate its clinical relevance, Fig. 2 displays the risk of adverse pathology by 4Kscore for men with biopsy Gleason 3 + 3 or 3 + 4, highlighting the relevance of the 4Kscore for decisions about the confirmatory biopsy and definitive treatment, respectively. The clinical performance of proceeding with a confirmatory biopsy among biopsy Gleason 3 + 3 men at various illustrative cut points is shown in Supplementary Table 8. For example, performing a confirmatory biopsy in men with a clinical + 4Kscore risk greater than 10% in 10,000 men would reduce the number of biopsies by 3086. Of these men avoiding confirmatory biopsy, 195/40 would have adverse pathology with/without ECE.

**Table 2:** Association between 4Kscore and adverse pathology on multivariable analysis, with optimism-corrected area under the curve (AUC)^a^.

Cohort	Sample size	Odds ratio^b^	95% CI^b^	*p* value^c^	Clinical	Clinical + 4Kscore	Only 4Kscore
*Primary analysis*^d^							
All biopsy Gleason Grades	2330	1.49	1.32,1.67	*p* < 0.0001	–	–	–
Biopsy Gleason Grade 3 + 3	1484	1.73	1.47,2.04	*p* < 0.0001	0.672	0.718	0.717
Biopsy Gleason Grade 3 + 4	524	1.32	1.10,1.59	*p* = 0.003	0.644	0.659	0.652
*Sensitivity analysis*^e^							
All biopsy Gleason Grades	1359	1.43	1.23,1.67	*p* < 0.0001	–	–	–
Biopsy Gleason Grade 3 + 3	789	1.55	1.27,1.89	*p* < 0.0001	0.651	0.724	0.724
Biopsy Gleason Grade 3 + 4	356	1.20	0.97,1.48	*p* = 0.10	0.677	0.686	0.632
*Sensitivity analysis*^f^							
All biopsy Gleason Grades	1359	1.29	1.10,1.52	*p* = 0.002	–	–	–
Biopsy Gleason Grade 3 + 3	789	1.64	1.33,2.03	*p* < 0.0001	0.625	0.753	0.760
Biopsy Gleason Grade 3 + 4	356	1.25	0.98,1.60	*p* = 0.073	0.616	0.642	0.636

^a^AUC for models in all biopsy Gleason Grades was not calculated since blood sample available prior to surgery was not representative of the distribution of all radical prostatectomy patients at Martini-Klinik.

^b^Odds ratios are for a one-point increase when taking the logit of the 4Kscore.

^c^*p* value presented from multivariable logistics in the corresponding analyses/cohort.

^d^Definition of adverse pathology includes ECE, and the clinical model consists of age, PSA, clinical stage and Gleason Grade on biopsy.

^e^As per primary analysis, except that the clinical model also includes the number of positive cores on biopsy, and tumour length on biopsy.

^f^As per primary analysis, except that the definition of adverse pathology excludes ECE, and the clinical model also includes the number of positive cores on biopsy, and tumour length on biopsy.

Among the 2064 patients with available BCR data, 395 men experienced BCR. The median follow-up time for those without BCR was 7.9 years (IQR 6.0, 9.1). The rate of salvage treatment within 1 year after surgery was 1.3% (95% CI 0.9%, 1.9%). Higher 4Kscore was associated with BCR on univariable analysis (HR 1.44; 95% CI 1.34, 1.54, *p* < 0.0001) and multivariable analysis after adjusting for preoperative clinical factors (HR 1.16, 95% CI 1.06, 1.26; *p* = 0.001). The association between 4Kscore and BCR was not significant after adjusting for post-operative variables (HR 1.00; 95% CI 0.90, 1.10; *p* = 0.9). Among men with biopsy Gleason 3 + 3 or 3 + 4 cancer, after adjusting for the preoperative nomogram, the 4Kscore was statistically significantly associated with BCR in men with biopsy Gleason 3 + 3 (HR 1.33; 95% CI 1.17, 1.52; *p* < 0.0001) but not in Gleason 3 + 4 (HR 1.09; 95% CI 0.92, 1.30; *p* = 0.3) (Table 3). Adding 4Kscore to the clinical model improved the prediction of BCR (C-index 0.630–0.660) within biopsy Gleason 3 + 3, but did not increase the C-index (0.620) among men with biopsy Gleason 3 + 4.

**Table 3:** Association between 4Kscore and BCR, after adjusting for the preoperative risk model with optimism-corrected C-index^a^.

	*N*	HR^b^	95% CI^b^	*p* value	Clinical	Clinical + 4Kscore
All biopsy Gleason Grades	2064	1.16	1.06, 1.26	0.001	–	–
Biopsy Gleason Grade 3 + 3	1324	1.33	1.17, 1.52	<0.0001	0.630	0.660
Biopsy Gleason Grade 3 + 4	468	1.09	0.92, 1.30	0.3	0.620	0.616

^a^AUC for the model in all biopsy Gleason Grades was not calculated since blood sample available prior to surgery was not representative of the distribution of all radical prostatectomy patients at Martini-Klinik.

^b^Hazard ratios are for a one-point increase when taking the logit of the 4Kscore.

## Discussion

We assessed whether the 4K panel (commercialised by OPKO Health Inc. as the 4Kscore test), helpful in detecting the presence of high-grade cancer within the prostate before a biopsy,^20^ could be expanded to help clinicians better predict the presence of adverse pathology within the prostate in men with biopsy grade 3 + 3 or 3 + 4 cancers. Such a tool could substantially improve decision-making by clinicians and patients with the decision of whether to have additional testing, such as confirmatory biopsy, prior to active surveillance (among men with biopsy grade 3 + 3 cancers), or to start active surveillance or have immediate radical surgery (among men with biopsy grade 3 + 4 cancers). We found that 4Kscore was strongly associated with both adverse pathology and BCR among men with biopsy Gleason 3 + 3, and improved the clinical utility of preoperative risk models across an appropriate range of risk thresholds. However, 4Kscore does not improve the value of post-operative risk models and, therefore, does not appear useful for counselling men after prostatectomy regarding the likelihood of recurrence. These findings support the use of the 4Kscore for biopsy decision-making as they suggest that, where grade 3 + 3 or 3 + 4 is to be found, the 4Kscore obtained at the time of biopsy decision-making could be used to make subsequent decisions about clinical management.

Our findings are supported by several prior studies. We have previously demonstrated that free PSA and hK2 enhance the predictive accuracy of clinical models predicting adverse pathology and BCR.^22–25^ In a cohort of 392 men from the Rotterdam arm of the European Randomized Study of Screening for Prostate Cancer treated with radical prostatectomy, predictions based on levels of four kallikrein markers accurately distinguished between pathologically insignificant and aggressive disease (addition of the kallikrein panel increased the AUC to 0.84, *p* < 0.0005).^26^ In a prospective multi-institutional study comprising 1312 men treated with radical prostatectomy at 26 sites in the United States, Punnen et al. showed that the 4Kscore was associated with Gleason score and ECE in the prostatectomy specimen. However, the 4Kscore was not found to improve the prediction of aggressive cancers when added to clinical prediction models, possibly due to small sample size.^27^

Regarding the utility of the 4Kscore in the active surveillance setting, Lin et al. prospectively evaluated 718 men enrolled in the multi-institutional Canary PASS trial, demonstrating that 4Kscore improved predictions of high-grade prostate cancer at confirmatory biopsy, but did not add substantive predictive value at subsequent surveillance biopsies.^28^ Similar findings were seen in a Spanish study of 137 men on active surveillance, where 4Kscore risk was associated with reclassification at confirmatory biopsy. Among men with 4Kscore below 7.5%, reclassification to Gleason 3 + 4 was missed in 2 men (6%) with no reclassification to Gleason 4 + 3.^29^

With 2330 patients, the present study is the largest series evaluating the role of the 4Kscore in predicting adverse pathology at radical prostatectomy. It is also the first study to evaluate the utility of the 4Kscore for the endpoint of BCR.

One limitation of this study is that detailed biopsy pathology with the number of positive cores and millimetres of cancerous tissue was available for 58% of the cohort. Moreover, the percentage of Gleason 4 was lacking in most Gleason 3 + 4 biopsies. As quantitative Gleason grading provides substantial prognostic information in Gleason 3 + 4 carcinomas,^30^ the impact of the 4Kscore may be dampened. However, the findings from sensitivity analyses that included detailed biopsy pathology data were similar to the main findings. This suggests that the 4Kscore adds important information about the risk of adverse pathology above and beyond that contained in detailed reporting of biopsy pathology, such as the number of cores and tumour length, which is not routinely reported by pathologists. A second possible limitation is that our study was restricted to a single centre. Our findings on Gleason 3 + 3, related to confirmatory biopsy, replicate those of a prior study;^28^ our findings on treatment decision-making in Gleason 3 + 4 disease require further investigation. Finally, our cohort and results are in the pre-MRI era, and the association between 4Kscore, outcomes and MRI is not fully established and requires further research.

## Conclusion

The 4Kscore strongly predicts adverse pathology and BCR in men with low-grade cancer on biopsy. In practice, the 4Kscore, along with additional tests such as MRI, could assist physicians and their patients in making the critical clinical decision for Gleason 3 + 3 cancers: whether to engage in additional risk assessment, such as a confirmatory biopsy, before initiating active surveillance. Evidence that the 4Kscore improves decision-making in biopsy Gleason 3 + 4 cancer (e.g. active surveillance vs definitive treatment) is less strong, but worthy of further study, especially in cohorts with low volume of Gleason pattern 4.

## Supplementary information

Supplementary Information

## Data Availability

The data sets analysed for this study will be made available to researchers on reasonable request to the corresponding author.

## References

[1] Schroder FH, Hugosson J, Roobol MJ, Tammela TL, Zappa M, Nelen V (2014). Screening and prostate cancer mortality: results of the European Randomised Study of Screening for Prostate Cancer (ERSPC) at 13 years of follow-up. Lancet.

[2] Ehdaie B, Vertosick E, Spaliviero M, Giallo-Uvino A, Taur Y, O’Sullivan M (2014). The impact of repeat biopsies on infectious complications in men with prostate cancer on active surveillance. J. Urol..

[3] Loeb S, Vellekoop A, Ahmed HU, Catto J, Emberton M, Nam R (2013). Systematic review of complications of prostate biopsy. Eur. Urol..

[4] Klotz L, Vesprini D, Sethukavalan P, Jethava V, Zhang L, Jain S (2015). Long-term follow-up of a large active surveillance cohort of patients with prostate cancer. J. Clin. Oncol..

[5] Kinsella N, Helleman J, Bruinsma S, Carlsson S, Cahill D, Brown C (2018). Active surveillance for prostate cancer: a systematic review of contemporary worldwide practices. Transl. Androl. Urol..

[6] Kaye DR, Qi J, Morgan TM, Linsell S, Ginsburg KB, Lane BR (2018). Pathological upgrading at radical prostatectomy for patients with Grade Group 1 prostate cancer: implications of confirmatory testing for patients considering active surveillance. BJU Int.

[7] Epstein JI, Feng Z, Trock BJ, Pierorazio PM (2012). Upgrading and downgrading of prostate cancer from biopsy to radical prostatectomy: incidence and predictive factors using the modified Gleason grading system and factoring in tertiary grades. Eur. Urol..

[8] Chun FK, Briganti A, Shariat SF, Graefen M, Montorsi F, Erbersdobler A (2006). Significant upgrading affects a third of men diagnosed with prostate cancer: predictive nomogram and internal validation. BJU Int.

[9] Reichard CA, Stephenson AJ, Klein EA (2015). Applying precision medicine to the active surveillance of prostate cancer. Cancer.

[10] Sternberg IA, Vela I, Scardino PT (2016). Molecular profiles of prostate cancer: to treat or not to treat. Annu Rev. Med..

[11] Parekh DJ, Punnen S, Sjoberg DD, Asroff SW, Bailen JL, Cochran JS (2015). A multi-institutional prospective trial in the USA confirms that the 4Kscore accurately identifies men with high-grade prostate cancer. Eur. Urol..

[12] Punnen S, Pavan N, Parekh DJ (2015). Finding the wolf in sheep’s clothing: the 4Kscore is a novel blood test that can accurately identify the risk of aggressive prostate cancer. Rev. Urol..

[13] Punnen S, Freedland SJ, Polascik TJ, Loeb S, Risk MC, Savage S (2018). A multi-institutional prospective trial confirms noninvasive blood test maintains predictive value in African American men. J. Urol..

[14] Vickers AJ, Vertosick EA, Sjoberg DD (2018). Value of a statistical model based on four kallikrein markers in blood, commercially available as 4Kscore, in all reasonable prostate biopsy subgroups. Eur. Urol..

[15] Sjoberg DD, Vickers AJ, Assel M, Dahlin A, Poon BY, Ulmert D (2018). Twenty-year risk of prostate cancer death by midlife prostate-specific antigen and a panel of four kallikrein markers in a large population-based cohort of healthy men. Eur. Urol..

[16] Stattin P, Vickers AJ, Sjoberg DD, Johansson R, Granfors T, Johansson M (2015). Improving the specificity of screening for lethal prostate cancer using prostate-specific antigen and a panel of kallikrein markers: a nested case-control study. Eur. Urol..

[17] Vickers AJ, Gupta A, Savage CJ, Pettersson K, Dahlin A, Bjartell A (2011). A panel of kallikrein marker predicts prostate cancer in a large, population-based cohort followed for 15 years without screening. Cancer Epidemiol. Biomark. Prev..

[18] Vaisanen V, Peltola MT, Lilja H, Nurmi M, Pettersson K (2006). Intact free prostate-specific antigen and free and total human glandular kallikrein 2. Elimination of assay interference by enzymatic digestion of antibodies to F(ab’)2 fragments. Anal. Chem..

[19] Brand TC, Zhang N, Crager MR, Maddala T, Dee A, Sesterhenn IA (2016). Patient-specific meta-analysis of 2 clinical validation studies to predict pathologic outcomes in prostate cancer using the 17-gene genomic prostate score. Urology.

[20] Bryant, R. J., Sjoberg, D. D., Vickers, A. J., Robinson, M. C., Kumar, R., Marsden L. et al. Predicting high-grade cancer at ten-core prostate biopsy using four kallikrein markers measured in blood in the ProtecT study. *J. Natl Cancer Inst.* **107**, djv095 (2015). 10.1093/jnci/djv095 PMC4554254 25863334

[21] Vickers AJ, Elkin EB (2006). Decision curve analysis: a novel method for evaluating prediction models. Med Decis. Mak..

[22] Wenske S, Korets R, Cronin AM, Vickers AJ, Fleisher M, Scher HI (2009). Evaluation of molecular forms of prostate-specific antigen and human kallikrein 2 in predicting biochemical failure after radical prostatectomy. Int J. Cancer.

[23] Haese A, Graefen M, Becker C, Noldus J, Katz J, Cagiannos I (2003). The role of human glandular kallikrein 2 for prediction of pathologically organ confined prostate cancer. Prostate.

[24] Haese A, Graefen M, Steuber T, Becker C, Pettersson K, Piironen T (2001). Human glandular kallikrein 2 levels in serum for discrimination of pathologically organ-confined from locally-advanced prostate cancer in total PSA-levels below 10 ng/ml. Prostate.

[25] Haese A, Becker C, Noldus J, Graefen M, Huland E, Huland H (2000). Human glandular kallikrein 2: a potential serum marker for predicting the organ confined versus non-organ confined growth of prostate cancer. J. Urol..

[26] Carlsson S, Maschino A, Schroder F, Bangma C, Steyerberg EW, van der Kwast T (2013). Predictive value of four kallikrein markers for pathologically insignificant compared with aggressive prostate cancer in radical prostatectomy specimens: results from the European Randomized Study of Screening for Prostate Cancer section Rotterdam. Eur. Urol..

[27] Punnen S, Nahar B, Prakash NS, Sjoberg DD, Zappala SM, Parekh DJ (2017). The 4Kscore predicts the grade and stage of prostate cancer in the radical prostatectomy specimen: results from a multi-institutional prospective trial. Eur. Urol. Focus.

[28] Lin DW, Newcomb LF, Brown MD, Sjoberg DD, Dong Y, Brooks JD (2017). Evaluating the four kallikrein panel of the 4Kscore for prediction of high-grade prostate cancer in men in the canary prostate active surveillance study. Eur. Urol..

[29] Borque-Fernando A, Rubio-Briones J, Esteban LM, Dong Y, Calatrava A, Gomez-Ferrer A (2018). Role of the 4Kscore test as a predictor of reclassification in prostate cancer active surveillance. Prostate Cancer Prostatic Dis..

[30] Sauter G, Steurer S, Clauditz TS, Krech T, Wittmer C, Lutz F (2016). Clinical utility of quantitative gleason grading in prostate biopsies and prostatectomy specimens. Eur. Urol..

