# Supplementary material for: A pre-specified model based on four kallikrein markers in blood improves predictions of adverse pathology and biochemical recurrence after radical prostatectomy
Source: Br J Cancer. 2020 May 29;123(4):604–9. doi: 10.1038/s41416-020-0914-7 (PMC7434907; doi:10.1038/s41416-020-0914-7)
Supplement: Supplementary file 1 — Supplementary Information [file 41416_2020_914_MOESM1_ESM.docx]

**SUPPLEMENTARY SECTION**

**METHODS**

As an exploratory analysis, we were interested in whether the association between the 4Kscore and the outcomes of adverse pathology and BCR, differed based on the biopsy tissue expression of five molecular markers: ERG, PTEN, EZH2, FOXA1, and HOXB13. The expression of five molecular markers in prostatectomy specimens: ERG, PTEN, EZH2, FOXA1, and HOXB13, was analyzed at the same institute as the kallikrein markers. We utilized two different multivariable regression models (logistic for adverse pathology, Cox for BCR) for each of the five markers of interest. The first model incorporated the main effect terms and an interaction term between the gene expression and the 4Kscore. The second model additionally included the predictors from the base model. While captured categorically, we dichotomized EZH2 (None vs Weak or Moderate or Strong), FOXA1 (None or Weak vs Moderate or Strong), and HOXB13 (None or Weak vs Moderate or Strong). ERG (positive) and PTEN (deletion) were assessed as they were captured.

**RESULTS**

**Supplementary Table 1** shows the distribution of the five molecular markers. The prevalence of missing gene expression data varies based on the molecular marker, ranging from a quarter (HOXB13) to a half (PTEN) missing. Among men with at least one marker measured, 30% of patients were missing at least two molecular markers. On multivariable analyses, there was no evidence of a difference in the relationship between the 4Kscore and adverse pathology or BCR based on gene expression (interaction terms between gene expression and the 4Kscore with a p-value > 0.05, **Supplementary Table 2 and 3, respectively**). Results were similar when assessed in the subgroup of Gleason Grade 3+3 patients (**Supplementary Table 4 and 5**).

**DISCUSSION**

As expression of KLK3/PSA and KLK2/hK2 is regulated by the androgen receptor and that mechanisms influencing androgen receptor signaling can influence levels and function of the kallikrein markers, we performed exploratory analyses of five different molecular tissue markers available for a subset of cases in our RP-cohort known to be associated with outcome, and selected on the basis that the tissue markers were either interacting with or regulated by the androgen receptor or frequently dysregulated in prostate cancer.

**Supplementary Table 1**. Frequency (proportion) of Molecular Marker data.

|  | N=2,330 |
| --- | --- |
| *ERG* (Positive) |  |
| No | 857 (37%) |
| Yes | 743 (32%) |
| Unknown | 730 (31%) |
| *PTEN* (Deletion) |  |
| No | 899 (39%) |
| Yes | 183 (7.9%) |
| Unknown | 1,248 (54%) |
| *EZH2* |  |
| Negative | 702 (30%) |
| Weak | 681 (29%) |
| Moderate | 170 (7.3%) |
| Strong | 12 (0.5%) |
| Unknown | 765 (33%) |
| *FOXA1* |  |
| Negative | 33 (1.4%) |
| Weak | 319 (14%) |
| Moderate | 697 (30%) |
| Strong | 398 (17%) |
| Unknown | 883 (38%) |
| *HOXB13* |  |
| Negative | 842 (36%) |
| Weak | 399 (17%) |
| Moderate | 361 (15%) |
| Strong | 164 (7.0%) |
| Unknown | 564 (24%) |

**Supplementary Table 2**. Multivariable analysis assessing association between 4Kscore and adverse pathology, after adjusting for the corresponding molecular marker, with an interaction term between 4Kscore and the corresponding molecular marker, with and without adjusting for variables in the clinical model.

|  |  | **Multivariable model with**  **4Kscore and the respective**  **molecular marker** | | | **Multivariable model with**  **4Kscore, respective**  **molecular marker,**  **and clinical model** | | |
| --- | --- | --- | --- | --- | --- | --- | --- |
|  | **N** | **OR*** | **95% C.I.*** | **p-value** | **OR*** | **95% C.I.*** | **p-value** |
|  | 1,600 |  |  |  |  |  |  |
| **4Kscore** |  | 1.65 | 1.45, 1.87 | <0.0001 | 1.27 | 1.09, 1.48 | 0.002 |
| **ERG (Positive)** |  | 1.20 | 0.90, 1.59 | 0.2 | 1.37 | 1.00, 1.89 | 0.051 |
| **Interaction:**  **4Kscore × ERG** |  | 1.14 | 0.94, 1.39 | 0.2 | 1.13 | 0.93, 1.39 | 0.2 |
|  |  |  |  |  |  |  |  |
|  | 1,082 |  |  |  |  |  |  |
| **4Kscore** |  | 1.73 | 1.53, 1.96 | <0.0001 | 1.23 | 1.04, 1.46 | 0.017 |
| **PTEN (Deletion)** |  | 3.15 | 2.03, 4.91 | <0.0001 | 3.72 | 2.18, 6.34 | <0.0001 |
| **Interaction:**  **4Kscore × PTENFISH** |  | 1.12 | 0.82, 1.53 | 0.5 | 1.37 | 0.96, 1.97 | 0.083 |
|  |  |  |  |  |  |  |  |
|  | 1,565 |  |  |  |  |  |  |
| **4Kscore** |  | 1.80 | 1.54, 2.10 | <0.0001 | 1.52 | 1.28, 1.82 | <0.0001 |
| **EZH2 (Weak or Moderate**  **or Strong)** |  | 1.58 | 1.19, 2.10 | 0.002 | 1.19 | 0.87, 1.64 | 0.3 |
| **Interaction:**  **4Kscore × EZH2** |  | 0.95 | 0.78, 1.17 | 0.6 | 0.90 | 0.73, 1.10 | 0.3 |
|  |  |  |  |  |  |  |  |
|  | 1,447 |  |  |  |  |  |  |
| **4Kscore** |  | 1.74 | 1.43, 2.11 | <0.0001 | 1.49 | 1.18, 1.89 | 0.001 |
| **FOXA1 (Moderate or**  **Strong)** |  | 1.89 | 1.37, 2.61 | 0.0001 | 1.78 | 1.23, 2.58 | 0.002 |
| **Interaction:**  **4Kscore × FOXA1** |  | 1.08 | 0.86, 1.35 | 0.5 | 0.99 | 0.78, 1.28 | >0.9 |
|  |  |  |  |  |  |  |  |
|  | 1,766 |  |  |  |  |  |  |
| **4Kscore** |  | 1.73 | 1.54, 1.94 | <0.0001 | 1.36 | 1.15, 1.60 | 0.0002 |
| **HOXB13 (Moderate**  **or Strong)** |  | 2.42 | 1.79, 3.27 | <0.0001 | 2.31 | 1.64, 3.24 | <0.0001 |
| **Interaction:**  **4Kscore × HOXB13** |  | 1.16 | 0.95, 1.42 | 0.14 | 1.19 | 0.96, 1.48 | 0.11 |

* Odds ratios are for a one-point increase when taking the logit of the 4Kscore.

**Supplementary Table 3**. Multivariable analysis assessing association between 4Kscore and BCR, after adjusting for the corresponding molecular marker, with an interaction term between 4Kscore and the corresponding molecular marker, with and without adjusting for preoperative risk.

|  |  | **Multivariable model with**  **4Kscore and the respective**  **molecular marker** | | | **Multivariable model with**  **4Kscore, respective**  **molecular marker,**  **and preoperative risk** | | |
| --- | --- | --- | --- | --- | --- | --- | --- |
|  | **N** | **HR*** | **95% C.I.*** | **p-value** | **HR*** | **95% C.I.*** | **p-value** |
|  | 1,413 |  |  |  |  |  |  |
| **4Kscore** |  | 1.33 | 1.21, 1.46 | <0.0001 | 1.08 | 0.96, 1.22 | 0.2 |
| **ERG (Positive)** |  | 1.00 | 0.77, 1.29 | >0.9 | 1.04 | 0.80, 1.35 | 0.8 |
| **Interaction:**  **4Kscore × ERG** |  | 1.15 | 0.98, 1.36 | 0.086 | 1.12 | 0.94, 1.32 | 0.2 |
|  |  |  |  |  |  |  |  |
|  | 948 |  |  |  |  |  |  |
| **4Kscore** |  | 1.40 | 1.28, 1.54 | <0.0001 | 1.12 | 0.99, 1.26 | 0.064 |
| **PTEN (Deletion)** |  | 2.57 | 1.88, 3.52 | <0.0001 | 2.50 | 1.81, 3.46 | <0.0001 |
| **Interaction:**  **4Kscore × PTENFISH** |  | 1.03 | 0.84, 1.25 | 0.8 | 1.04 | 0.85, 1.27 | 0.7 |
|  |  |  |  |  |  |  |  |
|  | 1,380 |  |  |  |  |  |  |
| **4Kscore** |  | 1.42 | 1.22, 1.64 | <0.0001 | 1.20 | 1.03, 1.39 | 0.017 |
| **EZH2 (Weak or Moderate**  **or Strong)** |  | 1.04 | 0.80, 1.36 | 0.8 | 0.87 | 0.66, 1.13 | 0.3 |
| **Interaction:**  **4Kscore × EZH2** |  | 1.02 | 0.85, 1.21 | 0.9 | 0.95 | 0.80, 1.12 | 0.5 |
|  |  |  |  |  |  |  |  |
|  | 1,284 |  |  |  |  |  |  |
| **4Kscore** |  | 1.61 | 1.29, 2.01 | <0.0001 | 1.27 | 0.98, 1.65 | 0.066 |
| **FOXA1 (Moderate or**  **Strong)** |  | 1.36 | 0.99, 1.86 | 0.056 | 1.28 | 0.92, 1.78 | 0.14 |
| **Interaction:**  **4Kscore × FOXA1** |  | 0.89 | 0.69, 1.13 | 0.3 | 0.88 | 0.68, 1.15 | 0.4 |
|  |  |  |  |  |  |  |  |
|  | 1,565 |  |  |  |  |  |  |
| **4Kscore** |  | 1.43 | 1.27, 1.61 | <0.0001 | 1.11 | 0.96, 1.28 | 0.2 |
| **HOXB13 (Moderate**  **or Strong)** |  | 1.60 | 1.24, 2.05 | 0.0003 | 1.61 | 1.24, 2.08 | 0.0003 |
| **Interaction:**  **4Kscore × HOXB13** |  | 0.99 | 0.84, 1.16 | 0.9 | 1.03 | 0.87, 1.21 | 0.8 |

* Hazard ratios are for a one-point increase when taking the logit of the 4Kscore.

**Supplementary Table 4**. Multivariable analysis assessing association between 4Kscore and adverse pathology, after adjusting for the corresponding molecular marker, with an interaction term between 4Kscore and the corresponding molecular marker, with and without adjusting for variables in the clinical model in Gleason Grade 3+3 patients.

|  |  | **Multivariable model with**  **4Kscore and the respective**  **molecular marker** | | | **Multivariable model with**  **4Kscore, respective**  **molecular marker,**  **and clinical model** | | |
| --- | --- | --- | --- | --- | --- | --- | --- |
|  | **N** | **OR*** | **95% C.I.*** | **p-value** | **OR*** | **95% C.I.*** | **p-value** |
|  | 965 |  |  |  |  |  |  |
| **4Kscore** |  | 1.72 | 1.41, 2.10 | <0.0001 | 1.54 | 1.21, 1.96 | 0.0004 |
| **ERG (Positive)** |  | 1.27 | 0.80, 2.01 | 0.3 | 1.32 | 0.82, 2.13 | 0.3 |
| **Interaction:**  **4Kscore × ERG** |  | 0.86 | 0.64, 1.15 | 0.3 | 0.91 | 0.68, 1.21 | 0.5 |
|  |  |  |  |  |  |  |  |
|  | 633 |  |  |  |  |  |  |
| **4Kscore** |  | 1.52 | 1.27, 1.80 | <0.0001 | 1.29 | 1.02, 1.64 | 0.036 |
| **PTEN (Deletion)** |  | 4.73 | 2.33, 9.60 | <0.0001 | 5.07 | 2.33, 11.02 | <0.0001 |
| **Interaction:**  **4Kscore × PTENFISH** |  | 1.56 | 0.92, 2.64 | 0.10 | 1.65 | 0.94, 2.88 | 0.081 |
|  |  |  |  |  |  |  |  |
|  | 959 |  |  |  |  |  |  |
| **4Kscore** |  | 1.62 | 1.32, 1.99 | <0.0001 | 1.64 | 1.31, 2.06 | <0.0001 |
| **EZH2 (Weak or Moderate**  **or Strong)** |  | 1.18 | 0.75, 1.83 | 0.5 | 1.12 | 0.71, 1.75 | 0.6 |
| **Interaction:**  **4Kscore × EZH2** |  | 1.01 | 0.75, 1.35 | >0.9 | 1.00 | 0.74, 1.33 | >0.9 |
|  |  |  |  |  |  |  |  |
|  | 862 |  |  |  |  |  |  |
| **4Kscore** |  | 1.67 | 1.32, 2.11 | <0.0001 | 1.69 | 1.33, 2.16 | <0.0001 |
| **FOXA1 (Moderate or**  **Strong)** |  | 1.19 | 0.73, 1.92 | 0.5 | 1.12 | 0.68, 1.82 | 0.7 |
| **Interaction:**  **4Kscore × FOXA1** |  | 1.02 | 0.76, 1.38 | 0.9 | 1.01 | 0.75, 1.36 | 0.9 |
|  |  |  |  |  |  |  |  |
|  | 1,091 |  |  |  |  |  |  |
| **4Kscore** |  | 1.62 | 1.39, 1.89 | <0.0001 | 1.62 | 1.35, 1.95 | <0.0001 |
| **HOXB13 (Moderate or**  **Strong)** |  | 1.86 | 1.16, 2.97 | 0.009 | 1.82 | 1.13, 2.93 | 0.013 |
| **Interaction:**  **4Kscore × HOXB13** |  | 1.38 | 1.01, 1.90 | 0.045 | 1.36 | 0.99, 1.87 | 0.057 |

* Odds ratios are for a one-point increase when taking the logit of the 4Kscore.

**Supplementary Table 5**. Multivariable analysis assessing association between 4Kscore and BCR, after adjusting for the corresponding molecular marker, with an interaction term between 4Kscore and the corresponding molecular marker, with and without adjusting for preoperative risk in Gleason Grade 3+3 patients.

|  |  | **Multivariable model with**  **4Kscore and the respective**  **molecular marker** | | | **Multivariable model with**  **4Kscore, respective**  **molecular marker,**  **and preoperative risk** | | |
| --- | --- | --- | --- | --- | --- | --- | --- |
|  | **N** | **HR*** | **95% C.I.*** | **p-value** | **HR*** | **95% C.I.*** | **p-value** |
|  | 856 |  |  |  |  |  |  |
| **4Kscore** |  | 1.34 | 1.13, 1.59 | 0.001 | 1.21 | 0.98, 1.49 | 0.074 |
| **ERG (Positive)** |  | 0.74 | 0.46, 1.20 | 0.2 | 0.73 | 0.46, 1.18 | 0.2 |
| **Interaction:**  **4Kscore × ERG** |  | 1.05 | 0.78, 1.42 | 0.7 | 1.04 | 0.77, 1.40 | 0.8 |
|  |  |  |  |  |  |  |  |
|  | 556 |  |  |  |  |  |  |
| **4Kscore** |  | 1.28 | 1.04, 1.57 | 0.020 | 1.24 | 0.96, 1.60 | 0.10 |
| **PTEN (Deletion)** |  | 3.45 | 1.84, 6.46 | 0.0001 | 3.40 | 1.74, 6.65 | 0.0003 |
| **Interaction:**  **4Kscore × PTENFISH** |  | 1.34 | 0.86, 2.10 | 0.2 | 1.34 | 0.83, 2.16 | 0.2 |
|  |  |  |  |  |  |  |  |
|  | 851 |  |  |  |  |  |  |
| **4Kscore** |  | 1.37 | 1.15, 1.62 | 0.0003 | 1.23 | 1.01, 1.49 | 0.036 |
| **EZH2 (Weak or Moderate**  **or Strong)** |  | 0.94 | 0.60, 1.47 | 0.8 | 0.91 | 0.57, 1.44 | 0.7 |
| **Interaction:**  **4Kscore × EZH2** |  | 1.13 | 0.84, 1.53 | 0.4 | 1.11 | 0.81, 1.51 | 0.5 |
|  |  |  |  |  |  |  |  |
|  | 770 |  |  |  |  |  |  |
| **4Kscore** |  | 1.48 | 1.12, 1.96 | 0.006 | 1.36 | 1.00, 1.86 | 0.051 |
| **FOXA1 (Moderate or**  **Strong)** |  | 1.31 | 0.78, 2.20 | 0.3 | 1.30 | 0.77, 2.19 | 0.3 |
| **Interaction:**  **4Kscore × FOXA1** |  | 0.97 | 0.69, 1.37 | 0.9 | 0.96 | 0.68, 1.36 | 0.8 |
|  |  |  |  |  |  |  |  |
|  | 974 |  |  |  |  |  |  |
| **4Kscore** |  | 0.97 | 0.69, 1.37 | 0.9 | 1.28 | 1.03, 1.59 | 0.025 |
| **HOXB13 (Moderate**  **or Strong)** |  | 1.43 | 0.89, 2.29 | 0.14 | 1.49 | 0.92, 2.42 | 0.11 |
| **Interaction:**  **4Kscore × HOXB13** |  | 1.08 | 0.79, 1.47 | 0.6 | 1.11 | 0.81, 1.52 | 0.5 |

* Hazard ratios are for a one-point increase when taking the logit of the 4Kscore.

**Supplementary Table 6a**. Association between 4Kscore and adverse pathology on multivariable analysis in men undergoing radical prostatectomy between 2002-2010.

|  | **Any 4Kscore** | | | | **4Kscore > 6.0%** | | | | **4Kscore > 7.5%** | | | |
| --- | --- | --- | --- | --- | --- | --- | --- | --- | --- | --- | --- | --- |
| **Cohort** | **Sample**  **Size** | **Odds**  **Ratio*** | **95% CI*** | **p-value** | **Sample**  **Size** | **Odds**  **Ratio*** | **95% CI*** | **p-value** | **Sample**  **Size** | **Odds**  **Ratio*** | **95% CI*** | **p-value** |
| (a) |  |  |  |  |  |  |  |  |  |  |  |  |
| All (Biopsy) Gleason Grades | 2,330 | 1.49 | 1.32,1.67 | p<0.0001 | 2,103 | 1.44 | 1.27,1.64 | p<0.0001 | 2,002 | 1.43 | 1.25,1.64 | p<0.0001 |
| (Biopsy) Gleason Grade 3+3 | 1,484 | 1.73 | 1.47,2.04 | p<0.0001 | 1,294 | 1.60 | 1.32,1.94 | p<0.0001 | 1,215 | 1.55 | 1.26,1.91 | p<0.0001 |
| (Biopsy) Gleason Grade 3+4 | 524 | 1.32 | 1.10,1.59 | p=0.003 | 497 | 1.30 | 1.08,1.57 | p=0.007 | 483 | 1.27 | 1.04,1.55 | p=0.017 |
| (b) |  |  |  |  |  |  |  |  |  |  |  |  |
| All (Biopsy) Gleason Grades | 1,359 | 1.43 | 1.23,1.67 | p<0.0001 | 1,243 | 1.36 | 1.15,1.61 | p=0.0003 | 1,189 | 1.34 | 1.12,1.60 | p=0.001 |
| (Biopsy) Gleason Grade 3+3 | 789 | 1.55 | 1.27,1.89 | p<0.0001 | 693 | 1.42 | 1.16,1.73 | p=0.001 | 651 | 1.39 | 1.13,1.70 | p=0.002 |
| (Biopsy) Gleason Grade 3+4 | 356 | 1.20 | 0.97,1.48 | p=0.10 | 340 | 1.18 | 0.94,1.48 | p=0.2 | 332 | 1.15 | 0.91,1.46 | p=0.2 |
| (c) |  |  |  |  |  |  |  |  |  |  |  |  |
| All (Biopsy) Gleason Grades | 2,330 | 1.38 | 1.21,1.58 | p<0.0001 | 2,103 | 1.39 | 1.21,1.61 | p<0.0001 | 2,002 | 1.42 | 1.22,1.64 | p<0.0001 |
| (Biopsy) Gleason Grade 3+3 | 1,484 | 1.90 | 1.53,2.36 | p<0.0001 | 1,294 | 1.92 | 1.52,2.43 | p<0.0001 | 1,215 | 1.91 | 1.50,2.43 | p<0.0001 |
| (Biopsy) Gleason Grade 3+4 | 524 | 1.35 | 1.09,1.68 | p=0.006 | 497 | 1.35 | 1.08,1.68 | p=0.008 | 483 | 1.32 | 1.05,1.66 | p=0.018 |
| (d) |  |  |  |  |  |  |  |  |  |  |  |  |
| All (Biopsy) Gleason Grades | 1,359 | 1.29 | 1.10,1.52 | p=0.002 | 1,243 | 1.29 | 1.09,1.53 | p=0.003 | 1,189 | 1.29 | 1.08,1.53 | p=0.005 |
| (Biopsy) Gleason Grade 3+3 | 789 | 1.64 | 1.33,2.03 | p<0.0001 | 693 | 1.54 | 1.23,1.92 | p=0.0001 | 651 | 1.54 | 1.23,1.92 | p=0.0002 |
| (Biopsy) Gleason Grade 3+4 | 356 | 1.25 | 0.98,1.60 | p=0.073 | 340 | 1.28 | 1.00,1.64 | p=0.052 | 332 | 1.23 | 0.95,1.60 | p=0.12 |

* Odds ratios are for a one-point increase when taking the logit of the 4Kscore.

(a) Definition of adverse pathology includes ECE, and the clinical model consists of age, PSA, clinical stage, Gleason Grade on biopsy.

(b) Definition of adverse pathology includes ECE, and the clinical model consists of age, PSA, clinical stage, Gleason Grade on biopsy, and additionally number of positive cores on biopsy, and tumor length on biopsy.

(c) Definition of adverse pathology excludes ECE, and the clinical model consists of age, PSA, clinical stage, Gleason Grade on biopsy.

(d) Definition of adverse pathology excludes ECE, and the clinical model consists of age, PSA, clinical stage, Gleason Grade on biopsy, and additionally number of positive cores on biopsy, and tumor length on biopsy.

**Supplementary Table 6b**. Association between 4Kscore and adverse pathology on multivariable analysis, incorporating TRUS volume, in men undergoing radical prostatectomy between 2002-2010.

|  | **Any 4Kscore** | | | | **4Kscore > 7.5%** | | | |
| --- | --- | --- | --- | --- | --- | --- | --- | --- |
| **Cohort** | **Sample**  **Size** | **Odds**  **Ratio*** | **95% CI*** | **p-value** | **Sample**  **Size** | **Odds**  **Ratio*** | **95% CI*** | **p-value** |
| (a) |  |  |  |  |  |  |  |  |
| All (Biopsy) Gleason Grades | 2,261 | 1.40 | 1.21,1.62 | p<0.0001 | 1,946 | 1.33 | 1.12,1.58 | p=0.001 |
| (Biopsy) Gleason Grade 3+3 | 1,431 | 1.59 | 1.27,2.00 | p<0.0001 | 1,174 | 1.43 | 1.11,1.86 | p=0.007 |
| (Biopsy) Gleason Grade 3+4 | 514 | 1.24 | 1.02,1.50 | p=0.028 | 473 | 1.16 | 0.94,1.43 | p=0.2 |
| (b) |  |  |  |  |  |  |  |  |
| All (Biopsy) Gleason Grades | 1,333 | 1.38 | 1.14,1.67 | p=0.001 | 1,168 | 1.28 | 1.03,1.61 | p=0.029 |
| (Biopsy) Gleason Grade 3+3 | 771 | 1.49 | 1.19,1.86 | p=0.001 | 638 | 1.34 | 1.07,1.68 | p=0.010 |
| (Biopsy) Gleason Grade 3+4 | 350 | 1.14 | 0.92,1.42 | p=0.2 | 326 | 1.09 | 0.85,1.39 | p=0.5 |
| (c) |  |  |  |  |  |  |  |  |
| All (Biopsy) Gleason Grades | 2,261 | 1.36 | 1.17,1.57 | p<0.0001 | 1,946 | 1.38 | 1.17,1.63 | p=0.0001 |
| (Biopsy) Gleason Grade 3+3 | 1,431 | 1.82 | 1.45,2.29 | p<0.0001 | 1,174 | 1.84 | 1.43,2.37 | p<0.0001 |
| (Biopsy) Gleason Grade 3+4 | 514 | 1.33 | 1.05,1.67 | p=0.016 | 473 | 1.29 | 1.00,1.65 | p=0.047 |
| (d) |  |  |  |  |  |  |  |  |
| All (Biopsy) Gleason Grades | 1,333 | 1.28 | 1.08,1.51 | p=0.004 | 1,168 | 1.28 | 1.06,1.54 | p=0.009 |
| (Biopsy) Gleason Grade 3+3 | 771 | 1.58 | 1.28,1.95 | p<0.0001 | 638 | 1.48 | 1.19,1.85 | p=0.001 |
| (Biopsy) Gleason Grade 3+4 | 350 | 1.24 | 0.96,1.60 | p=0.11 | 326 | 1.21 | 0.91,1.59 | p=0.2 |

* Odds ratios are for a one-point increase when taking the logit of the 4Kscore.

(a) Definition of adverse pathology includes ECE, and the clinical model consists of age, PSA, clinical stage, Gleason Grade on biopsy, and TRUS volume.

(b) Definition of adverse pathology includes ECE, and the clinical model consists of age, PSA, clinical stage, Gleason Grade on biopsy, TRUS volume and additionally number of positive cores on biopsy, and tumor length on biopsy.

(c) Definition of adverse pathology excludes ECE, and the clinical model consists of age, PSA, clinical stage, Gleason Grade on biopsy, and TRUS volume.

(d) Definition of adverse pathology excludes ECE, and the clinical model consists of age, PSA, clinical stage, Gleason Grade on biopsy, TRUS volume and additionally number of positive cores on biopsy, and tumor length on biopsy.

**Supplementary Table 6c**. Association between 4Kscore and adverse pathology on multivariable analysis in men undergoing radical prostatectomy between 2005 and 2010.

|  | **Any 4Kscore** | | | | **4Kscore > 7.5%** | | | |
| --- | --- | --- | --- | --- | --- | --- | --- | --- |
| **Cohort** | **Sample**  **Size** | **Odds**  **Ratio*** | **95% CI*** | **p-value** | **Sample**  **Size** | **Odds**  **Ratio*** | **95% CI*** | **p-value** |
| (a) |  |  |  |  |  |  |  |  |
| All (Biopsy) Gleason Grades | 1,522 | 1.48 | 1.29,1.70 | p<0.0001 | 1,325 | 1.40 | 1.21,1.63 | p<0.0001 |
| (Biopsy) Gleason Grade 3+3 | 894 | 1.68 | 1.38,2.05 | p<0.0001 | 736 | 1.49 | 1.19,1.86 | p=0.0004 |
| (Biopsy) Gleason Grade 3+4 | 369 | 1.33 | 1.07,1.67 | p=0.012 | 342 | 1.25 | 0.99,1.58 | p=0.062 |
| (b) |  |  |  |  |  |  |  |  |
| All (Biopsy) Gleason Grades | 939 | 1.42 | 1.19,1.70 | p=0.0001 | 831 | 1.29 | 1.07,1.57 | p=0.009 |
| (Biopsy) Gleason Grade 3+3 | 518 | 1.56 | 1.22,2.00 | p=0.0004 | 432 | 1.37 | 1.06,1.77 | p=0.015 |
| (Biopsy) Gleason Grade 3+4 | 252 | 1.23 | 0.93,1.61 | p=0.14 | 235 | 1.19 | 0.88,1.60 | p=0.3 |
| (c) |  |  |  |  |  |  |  |  |
| All (Biopsy) Gleason Grades | 1,522 | 1.40 | 1.20,1.63 | p<0.0001 | 1,325 | 1.39 | 1.18,1.64 | p=0.0001 |
| (Biopsy) Gleason Grade 3+3 | 894 | 1.78 | 1.33,2.39 | p=0.0001 | 736 | 1.69 | 1.23,2.33 | p=0.001 |
| (Biopsy) Gleason Grade 3+4 | 369 | 1.43 | 1.12,1.83 | p=0.005 | 342 | 1.38 | 1.07,1.78 | p=0.014 |
| (d) |  |  |  |  |  |  |  |  |
| All (Biopsy) Gleason Grades | 939 | 1.32 | 1.06,1.64 | p=0.013 | 831 | 1.28 | 1.01,1.61 | p=0.040 |
| (Biopsy) Gleason Grade 3+3 | 518 | 1.51 | 1.15,1.99 | p=0.003 | 432 | 1.38 | 1.03,1.84 | p=0.033 |
| (Biopsy) Gleason Grade 3+4 | 252 | 1.34 | 0.98,1.83 | p=0.068 | 235 | 1.32 | 0.96,1.82 | p=0.087 |

* Odds ratios are for a one-point increase when taking the logit of the 4Kscore.

(a) Definition of adverse pathology includes ECE, and the clinical model consists of age, PSA, clinical stage, Gleason Grade on biopsy.

(b) Definition of adverse pathology includes ECE, and the clinical model consists of age, PSA, clinical stage, Gleason Grade on biopsy, and additionally number of positive cores on biopsy, and tumor length on biopsy.

(c) Definition of adverse pathology excludes ECE, and the clinical model consists of age, PSA, clinical stage, Gleason Grade on biopsy.

(d) Definition of adverse pathology excludes ECE, and the clinical model consists of age, PSA, clinical stage, Gleason Grade on biopsy, and additionally number of positive cores on biopsy, and tumor length on biopsy.

**Supplementary Table 6d**. Association between 4Kscore and adverse pathology on multivariable analysis, incorporating TRUS volume, in men undergoing radical prostatectomy between 2005-2010.

|  | **Any 4Kscore** | | | | **4Kscore > 7.5%** | | | |
| --- | --- | --- | --- | --- | --- | --- | --- | --- |
| **Cohort** | **Sample**  **Size** | **Odds**  **Ratio*** | **95% CI*** | **p-value** | **Sample**  **Size** | **Odds**  **Ratio*** | **95% CI*** | **p-value** |
| (a) |  |  |  |  |  |  |  |  |
| All (Biopsy) Gleason Grades | 1,496 | 1.35 | 1.15,1.58 | p=0.0002 | 1,302 | 1.27 | 1.07,1.50 | p=0.007 |
| (Biopsy) Gleason Grade 3+3 | 875 | 1.55 | 1.21,1.97 | p=0.0005 | 720 | 1.39 | 1.06,1.82 | p=0.017 |
| (Biopsy) Gleason Grade 3+4 | 364 | 1.21 | 0.95,1.55 | p=0.12 | 337 | 1.10 | 0.85,1.43 | p=0.5 |
| (b) |  |  |  |  |  |  |  |  |
| All (Biopsy) Gleason Grades | 934 | 1.31 | 1.07,1.60 | p=0.009 | 827 | 1.19 | 0.96,1.48 | p=0.12 |
| (Biopsy) Gleason Grade 3+3 | 515 | 1.46 | 1.08,1.98 | p=0.014 | 430 | 1.31 | 0.96,1.78 | p=0.085 |
| (Biopsy) Gleason Grade 3+4 | 250 | 1.10 | 0.82,1.47 | p=0.5 | 233 | 1.04 | 0.75,1.43 | p=0.8 |
| (c) |  |  |  |  |  |  |  |  |
| All (Biopsy) Gleason Grades | 1,496 | 1.35 | 1.14,1.60 | p=0.0004 | 1,302 | 1.34 | 1.11,1.61 | p=0.002 |
| (Biopsy) Gleason Grade 3+3 | 875 | 1.60 | 1.19,2.15 | p=0.002 | 720 | 1.53 | 1.12,2.08 | p=0.008 |
| (Biopsy) Gleason Grade 3+4 | 364 | 1.41 | 1.08,1.85 | p=0.012 | 337 | 1.36 | 1.02,1.80 | p=0.034 |
| (d) |  |  |  |  |  |  |  |  |
| All (Biopsy) Gleason Grades | 934 | 1.27 | 1.01,1.60 | p=0.044 | 827 | 1.23 | 0.96,1.58 | p=0.10 |
| (Biopsy) Gleason Grade 3+3 | 515 | 1.37 | 1.04,1.80 | p=0.027 | 430 | 1.25 | 0.93,1.68 | p=0.14 |
| (Biopsy) Gleason Grade 3+4 | 250 | 1.27 | 0.90,1.80 | p=0.2 | 233 | 1.25 | 0.87,1.80 | p=0.2 |

* Odds ratios are for a one-point increase when taking the logit of the 4Kscore.

(a) Definition of adverse pathology includes ECE, and the clinical model consists of age, PSA, clinical stage, Gleason Grade on biopsy, and TRUS volume.

(b) Definition of adverse pathology includes ECE, and the clinical model consists of age, PSA, clinical stage, Gleason Grade on biopsy, TRUS volume and additionally number of positive cores on biopsy, and tumor length on biopsy.

(c) Definition of adverse pathology excludes ECE, and the clinical model consists of age, PSA, clinical stage, Gleason Grade on biopsy, and TRUS volume.

(d) Definition of adverse pathology excludes ECE, and the clinical model consists of age, PSA, clinical stage, Gleason Grade on biopsy, TRUS volume and additionally number of positive cores on biopsy, and tumor length on biopsy.

**Supplementary Table 7a**. Discrimination of various models in men undergoing radical prostatectomy between 2002-2010.

|  | **Any 4Kscore** | | | | **4Kscore > 6.0%** | | | | **4Kscore > 7.5%** | | | |
| --- | --- | --- | --- | --- | --- | --- | --- | --- | --- | --- | --- | --- |
| **Cohort** | **Sample**  **Size** | **Clinical** | **Clinical +**  **4Kscore** | **Only**  **4Kscore** | **Sample**  **Size** | **Clinical** | **Clinical +**  **4Kscore** | **Only**  **4Kscore** | **Sample**  **Size** | **Clinical** | **Clinical +**  **4Kscore** | **Only**  **4Kscore** |
| (a) |  |  |  |  |  |  |  |  |  |  |  |  |
| (Biopsy) Gleason Grade 3+3 | 1,484 | 0.672 | 0.718 | 0.717 | 1,294 | 0.659 | 0.686 | 0.683 | 1,215 | 0.651 | 0.675 | 0.672 |
| (Biopsy) Gleason Grade 3+4 | 524 | 0.644 | 0.659 | 0.652 | 497 | 0.626 | 0.642 | 0.633 | 483 | 0.630 | 0.640 | 0.626 |
| (b) |  |  |  |  |  |  |  |  |  |  |  |  |
| (Biopsy) Gleason Grade 3+3 | 789 | 0.651 | 0.724 | 0.724 | 693 | 0.626 | 0.688 | 0.684 | 651 | 0.616 | 0.670 | 0.672 |
| (Biopsy) Gleason Grade 3+4 | 356 | 0.677 | 0.686 | 0.632 | 340 | 0.680 | 0.686 | 0.618 | 332 | 0.673 | 0.674 | 0.612 |
| (c) |  |  |  |  |  |  |  |  |  |  |  |  |
| (Biopsy) Gleason Grade 3+3 | 1,484 | 0.716 | 0.768 | 0.766 | 1,294 | 0.722 | 0.762 | 0.763 | 1,215 | 0.718 | 0.762 | 0.758 |
| (Biopsy) Gleason Grade 3+4 | 524 | 0.646 | 0.667 | 0.667 | 497 | 0.640 | 0.659 | 0.656 | 483 | 0.640 | 0.653 | 0.648 |
| (d) |  |  |  |  |  |  |  |  |  |  |  |  |
| (Biopsy) Gleason Grade 3+3 | 789 | 0.625 | 0.753 | 0.760 | 693 | 0.614 | 0.719 | 0.725 | 651 | 0.617 | 0.721 | 0.727 |
| (Biopsy) Gleason Grade 3+4 | 356 | 0.616 | 0.642 | 0.636 | 340 | 0.615 | 0.639 | 0.633 | 332 | 0.615 | 0.634 | 0.621 |

(a) Definition of adverse pathology includes ECE, and the clinical model consists of age, PSA, clinical stage, Gleason Grade on biopsy.

(b) Definition of adverse pathology includes ECE, and the clinical model consists of age, PSA, clinical stage, Gleason Grade on biopsy, and additionally number of positive cores on biopsy, and tumor length on biopsy.

(c) Definition of adverse pathology excludes ECE, and the clinical model consists of age, PSA, clinical stage, Gleason Grade on biopsy.

(d) Definition of adverse pathology excludes ECE, and the clinical model consists of age, PSA, clinical stage, Gleason Grade on biopsy, and additionally number of positive cores on biopsy, and tumor length on biopsy.

**Supplementary Table 7b**. Discrimination of various models, incorporating TRUS volume, in men undergoing radical prostatectomy between 2002-2010.

|  | **Any 4Kscore** | | | | **4Kscore > 7.5%** | | | |
| --- | --- | --- | --- | --- | --- | --- | --- | --- |
| **Cohort** | **Sample**  **Size** | **Clinical** | **Clinical +**  **4Kscore** | **Only**  **4Kscore** | **Sample**  **Size** | **Clinical** | **Clinical +**  **4Kscore** | **Only**  **4Kscore** |
| (a) |  |  |  |  |  |  |  |  |
| (Biopsy) Gleason Grade 3+3 | 1,431 | 0.704 | 0.723 | 0.716 | 1,174 | 0.685 | 0.687 | 0.672 |
| (Biopsy) Gleason Grade 3+4 | 514 | 0.655 | 0.664 | 0.649 | 473 | 0.644 | 0.647 | 0.623 |
| (b) |  |  |  |  |  |  |  |  |
| (Biopsy) Gleason Grade 3+3 | 771 | 0.683 | 0.728 | 0.721 | 638 | 0.644 | 0.669 | 0.669 |
| (Biopsy) Gleason Grade 3+4 | 350 | 0.690 | 0.692 | 0.631 | 326 | 0.689 | 0.687 | 0.611 |
| (c) |  |  |  |  |  |  |  |  |
| (Biopsy) Gleason Grade 3+3 | 1,431 | 0.752 | 0.769 | 0.767 | 1,174 | 0.754 | 0.766 | 0.762 |
| (Biopsy) Gleason Grade 3+4 | 514 | 0.646 | 0.664 | 0.665 | 473 | 0.641 | 0.651 | 0.646 |
| (d) |  |  |  |  |  |  |  |  |
| (Biopsy) Gleason Grade 3+3 | 771 | 0.677 | 0.759 | 0.759 | 638 | 0.655 | 0.723 | 0.727 |
| (Biopsy) Gleason Grade 3+4 | 350 | 0.623 | 0.643 | 0.637 | 326 | 0.626 | 0.628 | 0.622 |

(a) Definition of adverse pathology includes ECE, and the clinical model consists of age, PSA, clinical stage, Gleason Grade on biopsy, and TRUS volume.

(b) Definition of adverse pathology includes ECE, and the clinical model consists of age, PSA, clinical stage, Gleason Grade on biopsy, TRUS volume and additionally number of positive cores on biopsy, and tumor length on biopsy.

(c) Definition of adverse pathology excludes ECE, and the clinical model consists of age, PSA, clinical stage, Gleason Grade on biopsy, and TRUS volume.

(d) Definition of adverse pathology excludes ECE, and the clinical model consists of age, PSA, clinical stage, Gleason Grade on biopsy, TRUS volume and additionally number of positive cores on biopsy, and tumor length on biopsy.

**Supplementary Table 7c**. Discrimination of various models in men undergoing radical prostatectomy between 2005 and 2010.

|  | **Any 4Kscore** | | | | **4Kscore > 7.5%** | | | |
| --- | --- | --- | --- | --- | --- | --- | --- | --- |
| **Cohort** | **Sample**  **Size** | **Clinical** | **Clinical +**  **4Kscore** | **Only**  **4Kscore** | **Sample**  **Size** | **Clinical** | **Clinical +**  **4Kscore** | **Only**  **4Kscore** |
| (a) |  |  |  |  |  |  |  |  |
| (Biopsy) Gleason Grade 3+3 | 894 | 0.696 | 0.729 | 0.725 | 736 | 0.670 | 0.687 | 0.679 |
| (Biopsy) Gleason Grade 3+4 | 369 | 0.645 | 0.665 | 0.655 | 342 | 0.634 | 0.639 | 0.621 |
| (b) |  |  |  |  |  |  |  |  |
| (Biopsy) Gleason Grade 3+3 | 518 | 0.687 | 0.734 | 0.738 | 432 | 0.645 | 0.676 | 0.683 |
| (Biopsy) Gleason Grade 3+4 | 252 | 0.655 | 0.661 | 0.632 | 235 | 0.646 | 0.647 | 0.608 |
| (c) |  |  |  |  |  |  |  |  |
| (Biopsy) Gleason Grade 3+3 | 894 | 0.750 | 0.778 | 0.774 | 736 | 0.723 | 0.743 | 0.743 |
| (Biopsy) Gleason Grade 3+4 | 369 | 0.635 | 0.658 | 0.659 | 342 | 0.631 | 0.640 | 0.632 |
| (d) |  |  |  |  |  |  |  |  |
| (Biopsy) Gleason Grade 3+3 | 518 | 0.713 | 0.788 | 0.793 | 432 | 0.683 | 0.735 | 0.749 |
| (Biopsy) Gleason Grade 3+4 | 252 | 0.594 | 0.620 | 0.621 | 235 | 0.595 | 0.600 | 0.600 |

(a) Definition of adverse pathology includes ECE, and the clinical model consists of age, PSA, clinical stage, Gleason Grade on biopsy.

(b) Definition of adverse pathology includes ECE, and the clinical model consists of age, PSA, clinical stage, Gleason Grade on biopsy, and additionally number of positive cores on biopsy, and tumor length on biopsy.

(c) Definition of adverse pathology excludes ECE, and the clinical model consists of age, PSA, clinical stage, Gleason Grade on biopsy.

(d) Definition of adverse pathology excludes ECE, and the clinical model consists of age, PSA, clinical stage, Gleason Grade on biopsy, and additionally number of positive cores on biopsy, and tumor length on biopsy.

**Supplementary Table 7d**. Discrimination of various models, incorporating TRUS volume, in men undergoing radical prostatectomy between 2005-2010.

|  | **Any 4Kscore** | | | | **4Kscore > 7.5%** | | | |
| --- | --- | --- | --- | --- | --- | --- | --- | --- |
| **Cohort** | **Sample**  **Size** | **Clinical** | **Clinical +**  **4Kscore** | **Only**  **4Kscore** | **Sample**  **Size** | **Clinical** | **Clinical +**  **4Kscore** | **Only**  **4Kscore** |
| (a) |  |  |  |  |  |  |  |  |
| (Biopsy) Gleason Grade 3+3 | 875 | 0.714 | 0.732 | 0.727 | 720 | 0.693 | 0.694 | 0.681 |
| (Biopsy) Gleason Grade 3+4 | 364 | 0.670 | 0.677 | 0.656 | 337 | 0.660 | 0.659 | 0.623 |
| (b) |  |  |  |  |  |  |  |  |
| (Biopsy) Gleason Grade 3+3 | 515 | 0.711 | 0.736 | 0.738 | 430 | 0.668 | 0.678 | 0.683 |
| (Biopsy) Gleason Grade 3+4 | 250 | 0.695 | 0.691 | 0.636 | 233 | 0.688 | 0.679 | 0.613 |
| (c) |  |  |  |  |  |  |  |  |
| (Biopsy) Gleason Grade 3+3 | 875 | 0.772 | 0.788 | 0.771 | 720 | 0.738 | 0.748 | 0.741 |
| (Biopsy) Gleason Grade 3+4 | 364 | 0.642 | 0.662 | 0.658 | 337 | 0.637 | 0.637 | 0.631 |
| (d) |  |  |  |  |  |  |  |  |
| (Biopsy) Gleason Grade 3+3 | 515 | 0.744 | 0.793 | 0.792 | 430 | 0.709 | 0.736 | 0.749 |
| (Biopsy) Gleason Grade 3+4 | 250 | 0.639 | 0.639 | 0.627 | 233 | 0.644 | 0.627 | 0.608 |

(a) Definition of adverse pathology includes ECE, and the clinical model consists of age, PSA, clinical stage, Gleason Grade on biopsy, and TRUS volume.

(b) Definition of adverse pathology includes ECE, and the clinical model consists of age, PSA, clinical stage, Gleason Grade on biopsy, TRUS volume and additionally number of positive cores on biopsy, and tumor length on biopsy.

(c) Definition of adverse pathology excludes ECE, and the clinical model consists of age, PSA, clinical stage, Gleason Grade on biopsy, and TRUS volume.

(d) Definition of adverse pathology excludes ECE, and the clinical model consists of age, PSA, clinical stage, Gleason Grade on biopsy, TRUS volume and additionally number of positive cores on biopsy, and tumor length on biopsy.

**Supplementary Table 8**. Clinical implication of proceeding with a confirmatory biopsy in GG 3+3 patients on respective models based on risk of adverse pathology.

|  |  |  | **Adverse Pathology** | | **Adverse Pathology,**  **excluding ECE** | |
| --- | --- | --- | --- | --- | --- | --- |
| **Biopsy**  **Threshold** | **Number**  **Biopsied** | **Biopsies**  **Avoided** | **Cases**  **Found** | **Cases**  **Missed** | **Cases**  **Found** | **Cases**  **Missed** |
| All Men | 10000 | 0 | 1597 | 0 | 512 | 0 |
| Clinical > 10% | 9858 | 142 | 1590 | 7 | 512 | 0 |
| Clinical > 15% | 3511 | 6489 | 842 | 755 | 323 | 189 |
| Clinical > 25% | 856 | 9144 | 297 | 1300 | 122 | 390 |
| Clinical > 35% | 249 | 9751 | 114 | 1483 | 47 | 465 |
| Clinical + 4Kscore > 10% | 6914 | 3086 | 1402 | 195 | 472 | 40 |
| Clinical + 4Kscore > 15% | 3841 | 6159 | 1024 | 573 | 397 | 115 |
| Clinical + 4Kscore > 25% | 1449 | 8551 | 532 | 1065 | 249 | 263 |
| Clinical + 4Kscore > 35% | 660 | 9340 | 303 | 1294 | 168 | 344 |
